# Supplementary material for: Literary runaway: Increasingly more references cited per academic research article from 1980 to 2019
Source: PLoS One. 2021 Aug 6;16(8):e0255849. doi: 10.1371/journal.pone.0255849 (PMC8345841; doi:10.1371/journal.pone.0255849)
Supplement: S1 File — (DOCX) [file pone.0255849.s001.docx]

Supporting Information for

**Literary runaway: Increasingly more references cited per academic research article from 1980 to 2019**

Can Dai, Quan Chen, Tao Wan, Fan Liu, Yanbing Gong, Qingfeng Wang^*^

*Corresponding author. Email: qfwang@wbgcas.cn

**This file includes:**

Tables S1 to S3

Table S1. Selection process for journals in eight chosen categories according to information in JCR year 2019 (updated June 29, 2020). Three criteria were used: (i) the journal title or type does not indicate its publishing preference for reviews; (ii) the publication frequency as indicated by JCR is greater than four issues per year (as of 2019); and (iii) the year of the inaugural issue is no later than 1998. Selected journals are shaded in gray.

| Category | Random integer | Journal name | Publication frequency  issues/year | First year of publication | Select?  Yes or No | Reason for rejection | Cumulative selected journal counts per category |
| --- | --- | --- | --- | --- | --- | --- | --- |
| Mathematics | 99 | Indiana University Mathematics Journal | 6 | 1952 | Yes |  | 1 |
| Mathematics | 237 | Order | 3 | 1984 | No | Contradicts Criterion 2 | 1 |
| Mathematics | 123 | Journal d’Analyse Mathématique | 3 | 1951 | No | Contradicts Criterion 2 | 1 |
| Mathematics | 301^a^ | Bulletin of Symbolic Logic | 4 | 1995 | No | Contradicts Criterion 2 | 1 |
| Mathematics | 160* | Sbornik: Mathematics | 12 | 1866 | Yes |  | 2 |
| Mathematics | 119 | Annali di Matematica Pura ed Applicata | 4 | 1850 | No | Contradicts Criterion 2 | 2 |
| Mathematics | 200 | Topological Methods in Nonlinear Analysis | 4 | 1993 | No | Contradicts Criterion 2 | 2 |
| Mathematics | 184 | Forum Mathematicum | 6 | 1989 | Yes |  | 3 |
| Mathematics | 86 | Analysis and Applications | 6 | 2003 | No | Contradicts Criterion 3 | 3 |
| Mathematics | 143 | Algebraic and Geometric Topology | 6 | 2001 | No | Contradicts Criterion 3 | 3 |
| Mathematics | 11 | Advances in Nonlinear Analysis | 4 | 2012 | No | Contradicts Criteria 2 & 3 | 3 |
| Mathematics | 59 | Proceedings of the London Mathematical Society | 12 | 1865 | Yes |  | 4 |
| Mathematics | 262 | Portugaliae Mathematica | 4 | 1937 | No | Contradicts Criterion 2 | 4 |
| Mathematics | 261^b^ | Ukrainian Mathematical Journal | 12 | 1949 | Yes |  | 5 |
| Mathematics | 95 | Communications on Pure and Applied Analysis | 6 | 2002 | No | Contradicts Criterion 3 | 5 |
| Mathematics | 164 | Ramanujan Journal | 9 | 1997 | Yes |  | 6 |
| Mathematics | 111 | Proceedings of the Royal Society of Edinburgh Section A – Mathematics | 6 | 1844 | Yes |  | 7 |
| Mathematics | 181 | Complex Analysis and Operator Theory | 8 | 2007 | No | Contradicts Criterion 3 | 7 |
| Mathematics | 66 | Bulletin of the American Mathematical Society | 4 | 1891 | No | Contradicts Criterion 2 | 7 |
| Mathematics | 84 | Combinatorica | 6 | 1981 | Yes |  | 8 |
| Mathematics | 113 | Tohoku Mathematical Journal | 4 | 1911 | No | Contradicts Criterion 2 | 8 |
| Mathematics | 20 | Journal of the European Mathematical Society | 6 | 1999 | No | Contradicts Criterion 3 | 8 |
| Mathematics | 31 | Analysis & PDE | 3 | 2008 | No | Contradicts Criteria 2 & 3 | 8 |
| Mathematics | 38 | Journal of Algebraic Geometry | 4 | 1992 | No | Contradicts Criterion 2 | 8 |
| Mathematics | 77 | Journal of Mathematical Analysis and Applications | 24 | 1960 | Yes |  | 9 |
| Mathematics | 207 | Zeitschrift für Analysis und ihre Anwendungen | 4 | 1982 | No | Contradicts Criterion 2 | 9 |
| Mathematics | 80 | Ergodic Theory and Dynamical Systems | 8 | 1981 | Yes |  | 10 |
| Education | 197* | Learning Culture and Social Interaction | 4 | 2012 | No | Contradicts Criteria 2 & 3 | 0 |
| Education | 200* | Teaching Sociology | 4 | 1973 | No | Contradicts Criterion 2 | 0 |
| Education | 14 | Sociology of Education | 4 | 1927 | No | Contradicts Criterion 2 | 0 |
| Education | 207* | Australian Journal of Education | 3 | 1957 | No | Contradicts Criterion 2 | 0 |
| Education | 89 | International Journal of Sustainability in Higher Education | 4 | 2000 | No | Contradicts Criteria 2 & 3 | 0 |
| Education | 203* | Innovations in Education and Teaching International | 6 | 1964 | Yes |  | 1 |
| Education | 164* | European Journal of Education | 4 | 1965 | No | Contradicts Criterion 2 | 1 |
| Education | 255* | Movimento | 4 | 2010 | No | Contradicts Criteria 2 & 3 | 1 |
| Education | 215* | Journal of Social Work Education | 4 | 1965 | No | Contradicts Criterion 2 | 1 |
| Education | 163* | International Journal for Academic Development | 4 | 1996 | No | Contradicts Criterion 2 | 1 |
| Education | 74* | Educational Administration Quarterly | 5 | 1965 | Yes |  | 2 |
| Education | 22 | Comunicar | 4 | 1993 | No | Contradicts Criterion 2 | 2 |
| Education | 36 | IEEE Transactions on Learning Technologies | 4 | 2008 | No | Contradicts Criteria 2 & 3 | 2 |
| Education | 56 | Assessment & Evaluation in Higher Education | 8 | 1975 | Yes |  | 3 |
| Education | 68 | Research in Higher Education | 8 | 1973 | Yes |  | 4 |
| Education | 233 | English Teaching-Practice and Critique | 3 | 2002 | No | Contradicts Criteria 2 & 3 | 4 |
| Education | 84 | TESOL Quarterly | 4 | 1967 | No | Contradicts Criterion 2 | 4 |
| Education | 225^c^ | Revista de Educación | 4 | 1940 | No | Contradicts Criterion 2 | 4 |
| Education | 33 | Higher Education | 12 | 1972 | Yes |  | 5 |
| Education | 29 | Journal of Education Policy | 6 | 1986 | Yes |  | 6 |
| Education | 37 | Metacognition and Learning | 3 | 2006 | No | Contradicts Criteria 2 & 3 | 6 |
| Education | 45 | Review of Higher Education | 4 | 1997 | No | Contradicts Criteria 1 & 2 | 6 |
| Education | 67 | Comparative Education Review | 4 | 1957 | No | Contradicts Criteria 1 & 2 | 6 |
| Education | 212* | Current Issues in Language Planning | 4 | 2000 | No | Contradicts Criteria 2 & 3 | 6 |
| Education | 82 | Minerva | 4 | 1962 | No | Contradicts Criterion 2 | 6 |
| Education | 244 | International Journal of Art & Design Education | 3 | 1982 | No | Contradicts Criterion 2 | 6 |
| Education | 25 | Educational Evaluation and Policy Analysis | 4 | 1979 | No | Contradicts Criterion 2 | 6 |
| Education | 147^d^ | International Journal of Science Education | 18 | 1979 | Yes |  | 7 |
| Education | 39 | Sport Education and Society | 8 | 1996 | Yes |  | 8 |
| Education | 186* | Journal of Economic Education | 4 | 1969 | No | Contradicts Criterion 2 | 8 |
| Education | 54 | International Journal of Management Education | 3 | 2000 | No | Contradicts Criteria 2 & 3 | 8 |
| Education | 30 | Studies in Higher Education | 12 | 1976 | Yes |  | 9 |
| Education | 72 | Journal of Educational Computing Research | 8 | 1985 | Yes |  | 10 |
| Management | 130 | Research in Transportation Business and Management | 4 | 2011 | No | Contradicts Criteria 2 & 3 | 0 |
| Management | 167^e^ | Journal of Marketing Management | 18 | 1985 | Yes |  | 1 |
| Management | 224 | Management & Organizational History | 4 | 2006 | No | Contradicts Criteria 2 & 3 | 1 |
| Management | 125 | Work Aging and Retirement | 4 | 2015 | No | Contradicts Criteria 2 & 3 | 1 |
| Management | 78 | Organization & Environment | 4 | 1987 | No | Contradicts Criterion 2 | 1 |
| Management | 119 | Journal of Sport Management | 4 | 1987 | No | Contradicts Criterion 2 | 1 |
| Management | 103 | MIT Sloan Management Review | 4 | 1959 | No | Contradicts Criteria 1 & 2 | 1 |
| Management | 121 | Management and Organization Review | 4 | 2005 | No | Contradicts Criteria 1, 2 & 3 | 1 |
| Management | 7 | Administrative Science Quarterly | 4 | 1956 | No | Contradicts Criterion 2 | 1 |
| Management | 136 | European Journal of International Management | 6 | 2007 | No | Contradicts Criterion 3 | 1 |
| Management | 76 | Industry and Innovation | 8 | 1993 | Yes |  | 2 |
| Management | 146* | Industrial and Corporate Change | 6 | 1992 | Yes |  | 3 |
| Management | 42 | Journal of Purchasing and Supply Management | 4 | 1994 | No | Contradicts Criterion 2 | 3 |
| Management | 206 | Engineering Management Journal | 4 | 1989 | No | Contradicts Criterion 2 | 3 |
| Management | 33* | Journal of Management Studies | 8 | 1964 | Yes |  | 4 |
| Management | 81 | Information and Organization | 4 | 1991 | No | Contradicts Criterion 2 | 4 |
| Management | 116 | Operations Research | 6 | 1952 | Yes |  | 5 |
| Management | 152* | Asia Pacific Journal of Human Resources | 4 | 1966 | No | Contradicts Criterion 2 | 5 |
| Management | 88 | British Journal of Management | 4 | 1990 | No | Contradicts Criterion 2 | 5 |
| Management | 149^f^ | Leadership & Organization Development Journal | 8 | 1980 | Yes |  | 6 |
| Management | 197 | International Journal of Emerging Markets | 4 | 2006 | No | Contradicts Criteria 2 & 3 | 6 |
| Management | 58* | Journal of Family Business Strategy | 4 | 2010 | No | Contradicts Criteria 2 & 3 | 6 |
| Management | 144* | Operations Management Research | 4 | 2008 | No | Contradicts Criteria 2 & 3 | 6 |
| Management | 65 | Tourism Management Perspectives | 4 | 2012 | No | Contradicts Criteria 2 & 3 | 6 |
| Management | 211 | Academia Revista Latinoamericana de Administración | 3 | 1988 | No | Contradicts Criterion 2 | 6 |
| Management | 73 | Journal of Hospitality and Tourism Management | 4 | 2002 | No | Contradicts Criteria 2 & 3 | 6 |
| Management | 126 | Journal of Nursing Management | 8 | 1993 | Yes |  | 7 |
| Management | 54 | Long Range Planning | 6 | 1968 | Yes |  | 8 |
| Management | 97 | International Journal of Forecasting | 4 | 1985 | No | Contradicts Criterion 2 | 8 |
| Management | 105 | Journal of Occupational and Organizational Psychology | 4 | 1975 | No | Contradicts Criterion 2 | 8 |
| Management | 225 | Negotiation Journal | 4 | 1985 | No | Contradicts Criterion 2 | 8 |
| Management | 178 | IMA Journal of Management Mathematics | 4 | 1986 | No | Contradicts Criterion 2 | 8 |
| Management | 208 | Science Technology and Society | 3 | 1996 | No | Contradicts Criterion 2 | 8 |
| Management | 210 | Canadian Journal of Administrative Sciences | 4 | 1984 | No | Contradicts Criterion 2 | 8 |
| Management | 120 | International Journal of Management Education | 3 | 2000 | No | Contradicts Criteria 2 & 3 | 8 |
| Management | 139* | International Journal of Accounting Information Systems | 4 | 2000 | No | Contradicts Criteria 2 & 3 | 8 |
| Management | 47 | Journal of Destination Marketing & Management | 4 | 2012 | No | Contradicts Criteria 2 & 3 | 8 |
| Management | 153* | Scandinavian Journal of Management | 4 | 1984 | No | Contradicts Criterion 2 | 8 |
| Management | 156* | Managerial Auditing Journal | 9 | 1986 | Yes |  | 9 |
| Management | 149* | Information Systems and E-Business Management | 4 | 2003 | No | Contradicts Criteria 2 & 3 | 9 |
| Management | 108 | Group & Organization Management | 6 | 1976 | Yes |  | 10 |
| Geosciences | 14 | Global Biogeochemical Cycles | 12 | 1987 | Yes |  | 1 |
| Geosciences | 21 | Catena | 12 | 1973 | Yes |  | 2 |
| Geosciences | 164 | Geoscientific Instrumentation Methods and Data Systems | 4 | 2012 | No | Contradicts Criteria 2 & 3 | 2 |
| Geosciences | 62 | Paleoceanography and Paleoclimatology | 12 | 1986 | Yes |  | 3 |
| Geosciences | 135 | Geodinamica Acta | 4 | 1987 | No | Contradicts Criterion 2 | 3 |
| Geosciences | 91 | Journal of Contaminant Hydrology | 12 | 1986 | Yes |  | 4 |
| Geosciences | 96 | Geoscience Letters | 1 | 2014 | No | Contradicts Criteria 2 & 3 | 4 |
| Geosciences | 118 | Geoarchaeology | 6 | 1986 | Yes |  | 5 |
| Geosciences | 108* | International Journal of Disaster Risk Science | 4 | 2010 | No | Contradicts Criteria 2 & 3 | 5 |
| Geosciences | 34 | Earth Surface Processes and Landforms | 15 | 1976 | Yes |  | 6 |
| Geosciences | 55 | Bulletin of Engineering Geology and the Environment | 4 | 1970 | No | Contradicts Criterion 2 | 6 |
| Geosciences | 112 | Journal of Palaeogeography | 1 | 2012 | No | Contradicts Criteria 2 & 3 | 6 |
| Geosciences | 139 | Swiss Journal of Geosciences | 3 | 1888 | No | Contradicts Criterion 2 | 6 |
| Geosciences | 33 | Natural Resources Research | 6 | 1992 | Yes |  | 7 |
| Geosciences | 127 | Natural Hazards Review | 4 | 2000 | No | Contradicts Criteria 1, 2 & 3 | 7 |
| Geosciences | 80 | Geosphere | 6 | 2005 | No | Contradicts Criterion 3 | 7 |
| Geosciences | 101 | Groundwater | 6 | 1963 | Yes |  | 8 |
| Geosciences | 32 | Geocarto International | 8 | 1986 | Yes |  | 9 |
| Geosciences | 120 | Quarterly Journal of Engineering Geology and Hydrogeology | 4 | 1967 | No | Contradicts Criterion 2 | 9 |
| Geosciences | 125 | International Journal of Speleology | 3 | 1964 | No | Contradicts Criterion 2 | 9 |
| Geosciences | 109 | Archaeological and Anthropological Sciences | 4 | 2009 | No | Contradicts Criteria 2 & 3 | 9 |
| Geosciences | 99 | Geomechanics and Geophysics for Geo-Energy and Geo-Resources | 4 | 2015 | No | Contradicts Criteria 2 & 3 | 9 |
| Geosciences | 70 | Journal of Archaeological Science | 12 | 1974 | Yes |  | 10 |
| Cell Biology | 167 | Journal of Muscle Research and Cell Motility | 6 | 1980 | Yes |  | 1 |
| Cell Biology | 9 | Science Translational Medicine | 5 | 2009 | No | Contradicts Criterion 3 | 1 |
| Cell Biology | 174 | Advances in Anatomy Embryology and Cell Biology | 4 | 1891 | No | Contradicts Criterion 2 | 1 |
| Cell Biology | 63 | Disease Models & Mechanisms | 12 | 2008 | No | Contradicts Criterion 3 | 1 |
| Cell Biology | 51 | American Journal of Respiratory Cell and Molecular Biology | 12 | 1989 | Yes |  | 2 |
| Cell Biology | 33 | EMBO Reports | 12 | 2000 | No | Contradicts Criterion 3 | 2 |
| Cell Biology | 23 | Plant Cell | 12 | 1989 | Yes |  | 3 |
| Cell Biology | 126 | Bioscience Reports | 6 | 1981 | Yes |  | 4 |
| Cell Biology | 130 | Connective Tissue Research | 6 | 1972 | Yes |  | 5 |
| Cell Biology | 106 | Cellular & Molecular Biology Letters | 1 | 1996 | No | Contradicts Criterion 2 | 5 |
| Cell Biology | 100* | Histopathology | 12 | 1977 | Yes |  | 6 |
| Cell Biology | 185 | Cell and Tissue Banking | 4 | 2000 | No | Contradicts Criteria 2 & 3 | 6 |
| Cell Biology | 178 | Methods in Cell Biology | 6 | 1964 | No | Contradicts Criterion 1 | 6 |
| Cell Biology | 151 | Acta Histochemica | 8 | 1954 | Yes |  | 7 |
| Cell Biology | 58 | FASEB Journal | 12 | 1987 | Yes |  | 8 |
| Cell Biology | 107 | Cellular Microbiology | 12 | 1999 | No | Contradicts Criterion 3 | 8 |
| Cell Biology | 11 | Molecular Cell | 24 | 1997 | Yes |  | 9 |
| Cell Biology | 57 | Cancer & Metabolism | 1 | 2013 | No | Contradicts Criterion 2 & 3 | 9 |
| Cell Biology | 50 | Current Opinion in Genetics & Development | 6 | 1991 | No | Contradicts Criterion 1 | 9 |
| Cell Biology | 116 | Inflammation Research | 12 | 1969 | Yes |  | 10 |
| Linguistics | 56 | Metaphor and Symbol | 4 | 1986 | No | Contradicts Criterion 2 | 0 |
| Linguistics | 11 | English for Specific Purposes | 4 | 1980 | No | Contradicts Criterion 2 | 0 |
| Linguistics | 103 | Narrative Inquiry | 2 | 1991 | No | Contradicts Criterion 2 | 0 |
| Linguistics | 132 | Hispania | 4 | 1917 | No | Contradicts Criterion 2 | 0 |
| Linguistics | 124 | World Englishes | 4 | 1981 | No | Contradicts Criterion 2 | 0 |
| Linguistics | 13 | Computational Linguistics | 4 | 1974 | No | Contradicts Criterion 2 | 0 |
| Linguistics | 63 | ELT Journal | 4 | 1946 | No | Contradicts Criterion 2 | 0 |
| Linguistics | 99 | Linguistics | 6 | 1963 | Yes |  | 1 |
| Linguistics | 117 | English Today | 4 | 1985 | No | Contradicts Criterion 2 | 1 |
| Linguistics | 141 | Language Problems & Language Planning | 3 | 1977 | No | Contradicts Criterion 2 | 1 |
| Linguistics | 8 | Studies in Second Language Acquisition | 4 | 1978 | No | Contradicts Criterion 2 | 1 |
| Linguistics | 169 | Dialectologia et Geolinguistica | 1 | 1993 | No | Contradicts Criterion 2 | 1 |
| Linguistics | 54 | Cognitive Linguistics | 4 | 1990 | No | Contradicts Criterion 2 | 1 |
| Linguistics | 158 | LEXIKOS | 1 | 1991 | No | Contradicts Criterion 2 | 1 |
| Linguistics | 156 | Text & Talk | 6 | 1981 | Yes |  | 2 |
| Linguistics | 144 | Studies in Language | 4 | 1977 | No | Contradicts Criterion 2 | 2 |
| Linguistics | 30 | Journal of Neurolinguistics | 4 | 1985 | No | Contradicts Criterion 2 | 2 |
| Linguistics | 4 | Language Teaching | 4 | 1968 | No | Contradicts Criterion 2 | 2 |
| Linguistics | 119 | Lingua | 12 | 1949 | Yes |  | 3 |
| Linguistics | 153 | Nordic Journal of Linguistics | 3 | 1978 | No | Contradicts Criterion 2 | 3 |
| Linguistics | 84 | Interaction Studies | 3 | 1997 | No | Contradicts Criterion 2 | 3 |
| Linguistics | 24 | International Journal of Multilingualism | 4 | 2004 | No | Contradicts Criteria 2 & 3 | 3 |
| Linguistics | 147 | International Journal of American Linguistics | 4 | 1917 | No | Contradicts Criterion 2 | 3 |
| Linguistics | 67 | Mind & Language | 5 | 1986 | Yes |  | 4 |
| Linguistics | 16 | Assessing Writing | 4 | 1994 | No | Contradicts Criterion 2 | 4 |
| Linguistics | 137^g^ | Linguistica Antverpiensia New Series – Themes in Translation Studies | 1 | 1967 | No | Contradicts Criterion 2 | 4 |
| Linguistics | 133 | International Journal of Lexicography | 4 | 1988 | No | Contradicts Criterion 2 | 4 |
| Linguistics | 178 | Languages | 4 | 1966 | No | Contradicts Criterion 2 | 4 |
| Linguistics | 25 | System | 4 | 1973 | No | Contradicts Criterion 2 | 4 |
| Linguistics | 136 | English Language & Linguistics | 3 | 1997 | No | Contradicts Criterion 2 | 4 |
| Linguistics | 151 | Journal of Germanic Linguistics | 4 | 1989 | No | Contradicts Criterion 2 | 4 |
| Linguistics | 27 | Language Cognition and Neuroscience | 10 | 1985 | Yes |  | 5 |
| Linguistics | 162 | Language and Linguistics | 4 | 2000 | No | Contradicts Criteria 2 & 3 | 5 |
| Linguistics | 187* | Language & History | 2 | 1984 | No | Contradicts Criterion 2 | 5 |
| Linguistics | 165 | Revue Française de Linguistique Appliquée | 2 | 2001 | No | Contradicts Criteria 2 & 3 | 5 |
| Linguistics | 116 | International Review of Applied Linguistics in Language Teaching | 4 | 1963 | No | Contradicts Criteria 1 & 2 | 5 |
| Linguistics | 145 | Porta Linguarum | 2 | 2004 | No | Contradicts Criteria 2 & 3 | 5 |
| Linguistics | 60 | Journal of Multilingual and Multicultural Development | 6 | 1980 | Yes |  | 6 |
| Linguistics | 6 | Language Learning | 4 | 1948 | No | Contradicts Criterion 2 | 6 |
| Linguistics | 179 | Poznan Studies in Contemporary Linguistics | 4 | 2011 | No | Contradicts Criteria 2 & 3 | 6 |
| Linguistics | 45 | Natural Language & Linguistic Theory | 4 | 1983 | No | Contradicts Criterion 2 | 6 |
| Linguistics | 155^h^ | International Journal of Speech Language and the Law | 2 | 1994 | No | Contradicts Criterion 2 | 6 |
| Linguistics | 47 | Second Language Research | 4 | 1985 | No | Contradicts Criterion 2 | 6 |
| Linguistics | 59 | Linguistic Inquiry | 4 | 1970 | No | Contradict Criterion 2 | 6 |
| Linguistics | 39 | Journal of Fluency Disorders | 4 | 1974 | No | Contradicts Criterion 2 | 6 |
| Linguistics | 182 | Language Matters | 3 | 1991 | No | Contradicts Criterion 2 | 6 |
| Linguistics | 22 | Language in Society | 5 | 1972 | Yes |  | 7 |
| Linguistics | 115 | English Teaching-Practice and Critique | 3 | 2002 | No | Contradicts Criteria 2 & 3 | 7 |
| Linguistics | 166 | Journal of African Languages and Linguistics | 2 | 1979 | No | Contradicts Criterion 2 | 7 |
| Linguistics | 123^i^ | Pragmatics | 4 | 1986 | No | Contradicts Criterion 2 | 7 |
| Linguistics | 130 | Journal of Comparative Germanic Linguistics | 3 | 1997 | No | Contradicts Criterion 2 | 7 |
| Linguistics | 35 | Aphasiology | 12 | 1987 | Yes |  | 8 |
| Linguistics | 82^j^ | Syntax | 4 | 1998 | No | Contradicts Criterion 2 | 8 |
| Linguistics | 97 | Interpreter and Translator Trainer | 3 | 2007 | No | Contradicts Criteria 2 & 3 | 8 |
| Linguistics | 36 | Linguistics and Philosophy | 6 | 1977 | Yes |  | 9 |
| Linguistics | 180 | Names – A Journal of Onomastics | 4 | 1953 | No | Contradicts Criterion 2 | 9 |
| Linguistics | 37 | Language Testing | 4 | 1984 | No | Contradicts Criterion 2 | 9 |
| Linguistics | 95 | First Language | 6 | 1980 | Yes |  | 10 |
| Ecology | 4 | Frontiers in Ecology and the Environment | 10 | 2003 | No | Contradicts Criterion 3 | 0 |
| Ecology | 9 | Methods in Ecology and Evolution | 12 | 2010 | No | Contradicts Criterion 3 | 0 |
| Ecology | 24 | Current Opinion in Insect Science | 6 | 2014 | No | Contradicts Criteria 1 & 3 | 0 |
| Ecology | 29 | Agriculture, Ecosystems & Environment | 18 | 1982 | Yes |  | 1 |
| Ecology | 25 | Journal of Animal Ecology | 6 | 1932 | Yes |  | 2 |
| Ecology | 6 | Ecology Letters | 12 | 1998 | Yes |  | 3 |
| Ecology | 139 | Journal of Natural History | 24 | 1838 | Yes |  | 4 |
| Ecology | 3 | Nature Ecology & Evolution | 12 | 2017 | No | Contradicts Criterion 3 | 4 |
| Ecology | 14 | Advances in Ecological Research | N/A | 1962 | No | Contradicts Criterion 1 | 4 |
| Ecology | 131 | Journal of Tropical Ecology | 6 | 1985 | Yes |  | 5 |
| Ecology | 56 | Journal of Vegetation Science | 6 | 1990 | Yes |  | 6 |
| Ecology | 110 | Web Ecology | 1 | 2000 | No | Contradicts Criteria 2 & 3 | 6 |
| Ecology | 135* | Rangeland Journal | 6 | 1976 | Yes |  | 7 |
| Ecology | 40 | Landscape Ecology | 10 | 1987 | Yes |  | 8 |
| Ecology | 60 | Applied Vegetation Science | 4 | 1998 | No | Contradicts Criterion 2 | 8 |
| Ecology | 134 | Evolutionary Ecology Research | 8 | 1999 | No | Contradicts Criterion 3 | 8 |
| Ecology | 84 | Biotropica | 6 | 1969 | Yes |  | 9 |
| Ecology | 150 | African Journal of Ecology | 4 | 1963 | No | Contradicts Criterion 2 | 9 |
| Ecology | 90 | Bulletin of the Peabody Museum of Natural History | 2 | 1925 | No | Contradicts Criterion 2 | 9 |
| Ecology | 50 | Mammal Review | 4 | 1970 | No | Contradicts Criteria 1 & 2 | 9 |
| Ecology | 48 | Ecosphere | 12 | 2010 | No | Contradicts Criterion 3 | 9 |
| Ecology | 80 | Oryx | 4 | 1950 | No | Contradicts Criterion 2 | 9 |
| Ecology | 157 | Northwest Science | 4 | 1927 | No | Contradicts Criterion 2 | 9 |
| Ecology | 163* | Vie et Milieu – Life and Environment | 4 | 1950 | No | Contradicts Criterion 2 | 9 |
| Ecology | 155 | Contemporary Problems of Ecology | 7 | 2008 | No | Contradicts Criterion 3 | 9 |
| Ecology | 95 | Journal of Plant Ecology | 6 | 2008 | No | Contradicts Criterion 3 | 9 |
| Ecology | 7 | Global Change Biology | 12 | 1995 | Yes |  | 10 |
| Computer | 22 | Information Processing & Management | 6 | 1963 | Yes |  | 1 |
| Computer | 153 | International Arab Journal of Information Technology | 6 | 2003 | No | Contradicts Criterion 3 | 1 |
| Computer | 40 | Information Systems Frontiers | 5 | 1999 | No | Contradicts Criterion 3 | 1 |
| Computer | 143 | Information Technology and Libraries | 4 | 1968 | No | Contradicts Criterion 2 | 1 |
| Computer | 76 | Optical Switching and Networking | 4 | 2005 | No | Contradicts Criteria 2 & 3 | 1 |
| Computer | 20 | IEEE Transactions on Knowledge and Data Engineering | 12 | 1989 | Yes |  | 2 |
| Computer | 65 | Peer-To-Peer Networking and Applications | 6 | 2008 | No | Contradicts Criterion 3 | 2 |
| Computer | 115 | Methods of Information in Medicine | 6 | 1962 | Yes |  | 3 |
| Computer | 39 | Ad Hoc Networks | 8 | 2003 | No | Contradicts Criterion 3 | 3 |
| Computer | 26 | IEEE Wireless Communications Letters | 6 | 2012 | No | Contradicts Criterion 3 | 3 |
| Computer | 84 | Journal of Network and Systems Management | 4 | 1993 | No | Contradicts Criterion 2 | 3 |
| Computer | 33 | IEEE Transactions on Network and Service Management | 4 | 2004 | No | Contradicts Criteria 2 & 3 | 3 |
| Computer | 145 | IEEE Latin America Transactions | 12 | 2003 | No | Contradicts Criterion 3 | 3 |
| Computer | 151 | KSII Transactions on Internet and Information Systems | 6 | 2007 | No | Contradicts Criterion 3 | 3 |
| Computer | 12 | MIS Quarterly | 4 | 1977 | No | Contradicts Criterion 2 | 3 |
| Computer | 9 | Information Sciences | 36 | 1968 | Yes |  | 4 |
| Computer | 156 | Bell Labs Technical Journal | 1 | 1922 | No | Contradicts Criterion 2 | 4 |
| Computer | 64 | Sustainable Computing-Informatics & Systems | 4 | 2011 | No | Contradicts Criteria 2 & 3 | 4 |
| Computer | 72 | Wireless Networks | 8 | 1995 | Yes |  | 5 |
| Computer | 67 | International Journal of Web and Grid Services | 4 | 2005 | No | Contradicts Criteria 2 & 3 | 5 |
| Computer | 107 | International Journal on Semantic Web and Information Systems | 4 | 2005 | No | Contradicts Criteria 2 & 3 | 5 |
| Computer | 6 | IEEE Transactions on Dependable and Secure Computing | 6 | 2004 | No | Contradicts Criterion 3 | 5 |
| Computer | 106 | Photonic Network Communications | 6 | 1999 | No | Contradicts Criterion 3 | 5 |
| Computer | 19 | IEEE Multimedia | 4 | 1994 | No | Contradicts Criterion 2 | 5 |
| Computer | 32 | Journal of Management Information Systems | 4 | 1984 | No | Contradicts Criterion 2 | 5 |
| Computer | 51 | Egyptian Informatics Journal | 3 | 2010 | No | Contradicts Criteria 2 & 3 | 5 |
| Computer | 4 | IEEE Network | 6 | 1987 | Yes |  | 6 |
| Computer | 130 | Journal of Organizational Computing and Electronic Commerce | 4 | 1991 | No | Contradicts Criterion 2 | 6 |
| Computer | 149 | Data Technologies and Applications | 4 | 1966 | No | Contradicts Criterion 2 | 6 |
| Computer | 93 | ACM Transactions on Knowledge Discovery from Data | 4 | 2007 | No | Contradicts Criteria 2 & 3 | 6 |
| Computer | 95 | ACM Transactions on Privacy and Security | 4 | 1998 | No | Contradicts Criterion 2 | 6 |
| Computer | 69 | Pervasive and Mobile Computing | 6 | 2005 | No | Contradicts Criterion 3 | 6 |
| Computer | 48 | ACM Transactions on Multimedia Computing Communications and Applications | 4 | 2005 | No | Contradicts Criteria 2 & 3 | 6 |
| Computer | 129 | International Journal of Distributed Sensor Networks | 12 | 2005 | No | Contradicts Criterion 3 | 6 |
| Computer | 49 | Semantic Web | 6 | 2010 | No | Contradicts Criterion 3 | 6 |
| Computer | 87 | Information Retrieval Journal | 6 | 1999 | No | Contradicts Criterion 3 | 6 |
| Computer | 74 | Mobile Networks & Applications | 6 | 1996 | Yes |  | 7 |
| Computer | 81^k^ | Journal of the Association for Information Science and Technology | 12 | 1950 | Yes |  | 8 |
| Computer | 5 | Computer Science Review | 4 | 2007 | No | Contradicts Criteria 1, 2 & 3 | 8 |
| Computer | 105 | Geoinformatica | 4 | 1997 | No | Contradicts Criterion 2 | 8 |
| Computer | 104 | ACM Transactions on Autonomous and Adaptive Systems | 4 | 2006 | No | Contradicts Criteria 2 & 3 | 8 |
| Computer | 54 | IEEE Transactions on Information Theory | 12 | 1953 | Yes |  | 9 |
| Computer | 119 | International Journal of Information Security | 6 | 2001 | No | Contradicts Criterion 3 | 9 |
| Computer | 71 | ACM Transactions on Intelligent Systems and Technology | 6 | 2010 | No | Contradicts Criterion 3 | 9 |
| Computer | 121 | International Journal of Sensor Networks | 12 | 2006 | No | Contradicts Criterion 3 | 9 |
| Computer | 73 | Data Mining and Knowledge Discovery | 6 | 1997 | Yes |  | 10 |

* indicates discrepancy in ranking order with updated JCR dataset in October, 2020.

a, two Mathematics journals tied at rank 301; first chosen

b, two Mathematics journals tied at rank 260; second chosen

c, two Education journals tied at rank 224; second chosen

d, two Education journals tied at rank 146; second chosen

e, two Management journals tied at rank 166; second chosen

f, two Management journals tied at rank 148; second chosen

g, two Linguistics journals tied at rank 136; second chosen

h, two Linguistics journals tied at rank 154; second chosen

i, two Linguistics journals tied at rank 122; second chosen

j, two Linguistics journals tied at rank 81; second chosen

k, two Computer Science journals tied at rank 80; second chosen

Table S2. Comparisons between journal-level and category-level reference densities (number of references per research article, mean ± s.e.) from 2003 to 2019. Most results indicated that there were no significant differences.

| Category | Year | Journal-level ratio | Category-level  ratio | One sample *t*-test | *P* |
| --- | --- | --- | --- | --- | --- |
| Mathematics | 2003 | 19.0 ± 0.9 | 16.1 | 3.32 | 0.01 |
|  | 2004 | 18.6 ± 1.1 | 16.8 | 1.59 | 0.15 |
|  | 2005 | 19.7 ± 1.6 | 16.9 | 1.74 | 0.12 |
|  | 2006 | 19.3 ± 1.0 | 17.6 | 1.72 | 0.12 |
|  | 2007 | 20.1 ± 1.1 | 17.9 | 2.00 | 0.08 |
|  | 2008 | 20.1 ± 1.2 | 18.3 | 1.58 | 0.15 |
|  | 2009 | 21.1 ± 1.1 | 19 | 1.84 | 0.10 |
|  | 2010 | 20.8 ± 1.7 | 19.3 | 0.84 | 0.42 |
|  | 2011 | 20.3 ± 1.9 | 19.8 | 0.26 | 0.80 |
|  | 2012 | 21.1 ± 1.6 | 20.3 | 0.51 | 0.63 |
|  | 2013 | 22.4 ± 1.4 | 20.9 | 1.08 | 0.31 |
|  | 2014 | 22.1 ± 1.4 | 21.2 | 0.65 | 0.53 |
|  | 2015 | 23.3 ± 1.5 | 21.9 | 0.94 | 0.37 |
|  | 2016 | 23.8 ± 1.4 | 22.5 | 0.91 | 0.39 |
|  | 2017 | 24.1 ± 1.6 | 23.1 | 0.65 | 0.53 |
|  | 2018 | 25.1 ± 1.7 | 23.9 | 0.73 | 0.48 |
|  | 2019 | 24.9 ± 1.6 | 25.1 | −0.16 | 0.88 |
| Education | 2003 | 38.2 ± 4.3 | 30.5 | 1.79 | 0.12 |
|  | 2004 | 39.4 ± 3.6 | 31.3 | 2.26 | 0.06 |
|  | 2005 | 41.7 ± 5.0 | 32.4 | 1.86 | 0.12 |
|  | 2006 | 43.7 ± 3.7 | 33.9 | 2.65 | 0.03 |
|  | 2007 | 43.9 ± 3.5 | 36.3 | 2.17 | 0.07 |
|  | 2008 | 44.5 ± 4.7 | 37.6 | 1.62 | 0.15 |
|  | 2009 | 43.3 ± 3.4 | 37.9 | 1.61 | 0.15 |
|  | 2010 | 44.8 ± 3.6 | 39.7 | 1.43 | 0.19 |
|  | 2011 | 46.4 ± 3.3 | 41.1 | 1.59 | 0.15 |
|  | 2012 | 49.7 ± 4.1 | 42.1 | 1.87 | 0.09 |
|  | 2013 | 48.3 ± 4.0 | 43.8 | 1.12 | 0.29 |
|  | 2014 | 49.1 ± 4.3 | 45.3 | 0.88 | 0.40 |
|  | 2015 | 49.9 ± 3.8 | 45.9 | 1.03 | 0.33 |
|  | 2016 | 52.9 ± 4.6 | 47.6 | 1.16 | 0.28 |
|  | 2017 | 56.0 ± 4.6 | 48.8 | 1.58 | 0.15 |
|  | 2018 | 53.3 ± 3.8 | 49.4 | 1.03 | 0.33 |
|  | 2019 | 55.9 ± 4.3 | 51.8 | 0.95 | 0.37 |
| Management | 2003 | 50.9 ± 9.2 | 37.9 | 1.42 | 0.29 |
|  | 2004 | 50.0 ± 8.5 | 38.9 | 1.30 | 0.29 |
|  | 2005 | 56.4 ± 6.1 | 41 | 2.51 | 0.09 |
|  | 2006 | 53.9 ± 7.6 | 44 | 1.30 | 0.29 |
|  | 2007 | 59.5 ± 4.3 | 45 | 3.41 | 0.04 |
|  | 2008 | 57.8 ± 7.9 | 46.2 | 1.47 | 0.24 |
|  | 2009 | 54.3 ± 8.2 | 46.4 | 0.96 | 0.39 |
|  | 2010 | 56.8 ± 7.4 | 54.5 | 0.31 | 0.77 |
|  | 2011 | 63.1 ± 8.8 | 54.7 | 0.95 | 0.38 |
|  | 2012 | 64.7 ± 7.6 | 58.3 | 0.85 | 0.43 |
|  | 2013 | 64.9 ± 6.5 | 60.4 | 0.70 | 0.51 |
|  | 2014 | 64.2 ± 6.3 | 63.9 | 0.04 | 0.97 |
|  | 2015 | 62.9 ± 6.0 | 64.4 | −0.25 | 0.81 |
|  | 2016 | 70.2 ± 7.9 | 66.8 | 0.42 | 0.68 |
|  | 2017 | 72.3 ± 6.4 | 67.8 | 0.70 | 0.50 |
|  | 2018 | 71.7 ± 6.9 | 69.5 | 0.32 | 0.76 |
|  | 2019 | 76.8 ± 7.9 | 70.7 | 0.77 | 0.46 |
| Geosciences | 2003 | 40.1 ± 3.5 | 26.1 | 4.04 | 0.01 |
|  | 2004 | 43.0 ± 2.5 | 34.5 | 3.42 | 0.02 |
|  | 2005 | 43.5 ± 3.7 | 35.8 | 2.08 | 0.09 |
|  | 2006 | 45.8 ± 2.6 | 37.8 | 3.10 | 0.03 |
|  | 2007 | 45.9 ± 2.3 | 38.4 | 3.31 | 0.02 |
|  | 2008 | 47.6 ± 1.5 | 39.3 | 5.45 | 0.00 |
|  | 2009 | 48.4 ± 1.5 | 39.6 | 5.75 | 0.00 |
|  | 2010 | 52.7 ± 2.1 | 46.1 | 3.16 | 0.03 |
|  | 2011 | 55.6 ± 3.4 | 46.9 | 2.60 | 0.05 |
|  | 2012 | 54.2 ± 4.1 | 48.9 | 1.30 | 0.24 |
|  | 2013 | 54.4 ± 3.2 | 51.6 | 0.89 | 0.41 |
|  | 2014 | 54.2 ± 4.3 | 53.4 | 0.17 | 0.87 |
|  | 2015 | 57.2 ± 5.1 | 55 | 0.44 | 0.67 |
|  | 2016 | 57.3 ± 5.4 | 55 | 0.43 | 0.68 |
|  | 2017 | 58.6 ± 5.0 | 56.9 | 0.35 | 0.74 |
|  | 2018 | 65.1 ± 5.1 | 59.3 | 1.12 | 0.29 |
|  | 2019 | 66.3 ± 5.1 | 59.4 | 1.35 | 0.21 |
| Cell Biology | 2003 | 37.7 ± 3.0 | 40.3 | −0.87 | 0.41 |
|  | 2004 | 39.5 ± 3.1 | 41.3 | −0.59 | 0.57 |
|  | 2005 | 39.9 ± 3.2 | 41.7 | −0.55 | 0.60 |
|  | 2006 | 38.8 ± 3.3 | 42 | −0.96 | 0.36 |
|  | 2007 | 40.5 ± 3.4 | 42.3 | −0.54 | 0.60 |
|  | 2008 | 40.7 ± 3.3 | 43 | −0.69 | 0.51 |
|  | 2009 | 41.9 ± 2.9 | 43.6 | −0.59 | 0.57 |
|  | 2010 | 43.2 ± 3.2 | 45 | −0.57 | 0.59 |
|  | 2011 | 43.6 ± 3.3 | 46.1 | −0.86 | 0.41 |
|  | 2012 | 44.3 ± 3.2 | 46.5 | −0.69 | 0.51 |
|  | 2013 | 45.1 ± 3.3 | 46.6 | −0.46 | 0.66 |
|  | 2014 | 43.2 ± 3.8 | 47 | −1.01 | 0.34 |
|  | 2015 | 45.1 ± 3.2 | 47.1 | −0.64 | 0.54 |
|  | 2016 | 46.7 ± 3.6 | 47.5 | −0.21 | 0.84 |
|  | 2017 | 49.0 ± 3.8 | 50.1 | −0.28 | 0.79 |
|  | 2018 | 48.3 ± 3.9 | 50.1 | −0.47 | 0.65 |
|  | 2019 | 51.4 ± 4.6 | 50.3 | 0.24 | 0.82 |
| Linguistics | 2006 | 43.6 ± 2.3 | 45.2 | −0.70 | 0.52 |
|  | 2007 | 46.5 ± 2.5 | 45.3 | 0.47 | 0.67 |
|  | 2008 | 44.1 ± 3.0 | 44.3 | −0.08 | 0.94 |
|  | 2009 | 45.0 ± 3.0 | 43.6 | 0.48 | 0.65 |
|  | 2010 | 45.2 ± 5.2 | 46.2 | −0.19 | 0.86 |
|  | 2011 | 47.7 ± 4.0 | 46.5 | 0.31 | 0.77 |
|  | 2012 | 51.0 ± 3.2 | 48.9 | 0.68 | 0.52 |
|  | 2013 | 51.3 ± 3.8 | 49 | 0.61 | 0.57 |
|  | 2014 | 50.1 ± 3.1 | 49.2 | 0.31 | 0.77 |
|  | 2015 | 57.4 ± 3.8 | 50.8 | 1.76 | 0.12 |
|  | 2016 | 55.2 ± 2.8 | 50.8 | 1.56 | 0.16 |
|  | 2017 | 53.7 ± 2.5 | 51.8 | 0.77 | 0.46 |
|  | 2018 | 56.4 ± 2.4 | 53.5 | 1.20 | 0.26 |
|  | 2019 | 59.0 ± 2.2 | 54.1 | 2.22 | 0.05 |
| Ecology | 2003 | 43.1 ± 2.2 | 41.2 | 0.89 | 0.40 |
|  | 2004 | 42.8 ± 2.2 | 42.5 | 0.13 | 0.90 |
|  | 2005 | 44.1 ± 2.2 | 43.3 | 0.38 | 0.71 |
|  | 2006 | 44.4 ± 2.2 | 44.4 | −0.02 | 0.98 |
|  | 2007 | 47.2 ± 1.8 | 44.4 | 1.55 | 0.16 |
|  | 2008 | 48.1 ± 2.1 | 45.2 | 1.38 | 0.20 |
|  | 2009 | 49.0 ± 2.5 | 46.4 | 1.05 | 0.32 |
|  | 2010 | 51.0 ± 2.2 | 50.5 | 0.24 | 0.81 |
|  | 2011 | 53.0 ± 2.4 | 51.8 | 0.46 | 0.66 |
|  | 2012 | 51.3 ± 2.5 | 52.1 | −0.31 | 0.76 |
|  | 2013 | 53.4 ± 2.5 | 53.9 | −0.20 | 0.85 |
|  | 2014 | 54.0 ± 2.7 | 55 | −0.37 | 0.72 |
|  | 2015 | 55.0 ± 2.8 | 56.1 | −0.39 | 0.71 |
|  | 2016 | 59.2 ± 2.5 | 58.2 | 0.39 | 0.70 |
|  | 2017 | 60.6 ± 2.8 | 59.7 | 0.31 | 0.76 |
|  | 2018 | 61.9 ± 2.7 | 60.6 | 0.49 | 0.63 |
|  | 2019 | 62.8 ± 3.2 | 62 | 0.25 | 0.81 |
| Computer Science | 2003 | 24.2 ± 1.8 | 23.2 | 0.58 | 0.58 |
|  | 2004 | 22.9 ± 1.6 | 23.8 | −0.53 | 0.61 |
|  | 2005 | 25.1 ± 1.9 | 25.3 | −0.11 | 0.92 |
|  | 2006 | 25.6 ± 2.0 | 25.9 | −0.15 | 0.89 |
|  | 2007 | 26.8 ± 2.1 | 27.2 | −0.20 | 0.84 |
|  | 2008 | 27.3 ± 2.2 | 27.7 | −0.18 | 0.86 |
|  | 2009 | 28.6 ± 2.4 | 28.6 | −0.02 | 0.99 |
|  | 2010 | 29.6 ± 2.6 | 30.7 | −0.42 | 0.69 |
|  | 2011 | 32.7 ± 3.3 | 32.4 | 0.09 | 0.93 |
|  | 2012 | 32.4 ± 3.5 | 32.3 | 0.04 | 0.97 |
|  | 2013 | 33.7 ± 3.1 | 34.4 | −0.21 | 0.84 |
|  | 2014 | 36.6 ± 3.4 | 35.8 | 0.24 | 0.82 |
|  | 2015 | 39.0 ± 4.3 | 36.5 | 0.58 | 0.58 |
|  | 2016 | 38.6 ± 3.3 | 37.2 | 0.43 | 0.68 |
|  | 2017 | 39.7 ± 4.2 | 37.8 | 0.46 | 0.66 |
|  | 2018 | 38.7 ± 3.9 | 38.6 | 0.03 | 0.97 |
|  | 2019 | 40.6 ± 4.1 | 39.3 | 0.31 | 0.76 |

Table S3. Correlations among number of references per research article, page length and year of publication (1980–2002) in eight disciplinary categories.

| Category | Sample size | Number of references and page length | | Number of references and year | | Page length and year | |
| --- | --- | --- | --- | --- | --- | --- | --- |
|  |  | Pearson *r* | Spearman *ρ* | Pearson *r* | Spearman *ρ* | Pearson *r* | Spearman *ρ* |
| Mathematics | 2020 | 0.498*** | 0.508*** | 0.198*** | 0.207*** | 0.183*** | 0.192*** |
| Education | 2030 | 0.544*** | 0.564*** | 0.299*** | 0.332*** | 0.250*** | 0.260*** |
| Management | 1800 | 0.665*** | 0.680*** | 0.355*** | 0.382*** | 0.249*** | 0.253*** |
| Geosciences | 1870 | 0.465*** | 0.529*** | 0.245*** | 0.267*** | 0.108*** | 0.122*** |
| Cell Biology | 1870 | 0.417*** | 0.410*** | 0.358*** | 0.372*** | −0.011 | 0.050* |
| Linguistics | 2360 | 0.479*** | 0.519*** | 0.298*** | 0.315*** | 0.166*** | 0.199*** |
| Ecology | 1720 | 0.307*** | 0.365*** | 0.321*** | 0.333*** | 0.067** | 0.093*** |
| Computer Science | 1660 | 0.312*** | 0.370*** | 0.214*** | 0.260*** | 0.198*** | 0.249*** |

* 0.01< *P* < 0.05

** 0.001< *P* < 0.01

*** *P* < 0.001
