# Supplementary material for: Literary runaway: Increasingly more references cited per academic research article from 1980 to 2019
Source: PLoS One. 2021 Aug 6;16(8):e0255849. doi: 10.1371/journal.pone.0255849 (PMC8345841; doi:10.1371/journal.pone.0255849)
Supplement: Attachment — Submitted filename: Response to reviewers.docx [file pone.0255849.s002.docx]

Review Comments to the Author

Please use the space provided to explain your answers to the questions above. You may also include additional comments for the author, including concerns about dual publication, research ethics, or publication ethics. (Please upload your review as an attachment if it exceeds 20,000 characters)

Reviewer #1: I appreciate the work done by all the autors. I do not find any short comings in the manuscript. First of all, the literature study is comprehensive and it covers the said topic. The language used is appropriate and upto the mark.

*Response: We appreciate the reviewer’s positive evaluation of our work. It is very encouraging. Thanks!*

Reviewer #2: The manuscript "Literary runaway: Increasingly more references cited per academic research article from 1980 to 2019" presents an analysis of the average number of references in scientific citable papers. Eight subject categories are sampled for the time period 1980-2003 and thirty subject categories are analyzed for two years (2003 and 2019). Statistical analyses show an overall increasing number of references per paper on average over the years. Overall, the manuscript is well written and should be of interest to the readership of PLoS One. However, some improvements should be made before I can recommend publication of this manuscript.

The data are analyzed using R, and the authors pledge to make the data available upon acceptance. For the increasing benefit of the readers and to help reproducing the results, I would appreciate if the authors would share their R code with the readers.

*Response: Thanks for the reviewer’s suggestion. We have already uploaded the original datasets and R codes (both statistics and graphing) to Figshare Digital Repository. DOI: 10.6084/m9.figshare.14811363. They can also be accessed through the shared link* [*https://figshare.com/s/5da4b6135d01476cb49c*](https://figshare.com/s/5da4b6135d01476cb49c)

I wonder if the authors could provide confidence intervals in the plots of Fig. 2.

*Response: We had made an alternative figure with confidence intervals (see below). However, we think the interval overlapping somehow made the figure greyish and the regression lines not as sharp. Plus, the confidence intervals are generally small and r-squares high for all regressions. Thus, we prefer to report a cleaner figure.*

Two of the sampled subject categories are Mathematics and Linguistics. These subject categories should be regarded as problematic for bibliometric treatment. A discussion about this is warranted.

*Response: Thanks for pointing out the particularity of the two disciplines. We have now added some discussion regarding this limitation (Line 349-352).*

The authors analyze all cited references and also cite previous work that analyzed all cited references in papers as far as I can see. DOI 10.1016/j.joi.2016.07.002 also investigated the growth of linked cited references within a three-year window. This might be interesting in this context as it shows that the increasing bulk of previous knowledge plus the recent knowledge is not the reason for the observed trend. Only the references to the recent knowledge are increasing on average over time, too.

*Response: We enjoyed reading the reference mentioned (Bornmann & Haunschild 2016) and found it was quite relevant. The growth of linked cited references from 1985 to 2014 suggested that authors indeed cited more recent literature (within 3 years of publication) across all fields. Among the eight categories in our study, when we bounded reference counts to the same time frame, most disciplines had an increase of more than 20 references (Fig 2). However, we were surprised to find that the magnitude of increase was a lot lower in Bornmann & Haunschild 2016, with roughly 1-6 references. Such a disparity exactly pointed out that researchers disproportionately cited more old papers than recent ones, which echoed our discussion nicely. As we were thinking adding this work to existing explanations, we found it required extensive logical elaborations and it was not as straightforward as the studies that aimed at addressing the same hypothesis. Therefore, we left the discussion as it was. Thanks for directing an awesome study to us though, and we also like its idea of normalizing citation scores.*

Did the authors check if the average number of references is increasing for it is corrected for the increasing number of papers per year?

*Response: We are not sure what analysis the reviewer referred to exactly. We tried to conduct multiple regressions with year and total number of articles as independent variables of the eight chosen categories. Interestingly, the number of papers published was either not a significant source or showed up as a negative impact on reference density. Regardless, “year” always positively affected reference counts. We think it is due to the fact that the increase in publication is a lot steeper than that in reference number. Therefore, we have adopted the methodology that other literature used, accounting for this problem as an exponential growth instead of linear. It is essentially another way of correction based on the starting point of publications or reference density on yearly basis. The significant correlation indicated that the magnitude of increase in reference density echoed that in total publication among disciplinary categories. However, as we already emphasized in discussion, the increasing trend in reference density cannot be adequately explained by the sole factor of publication.*

The last paragraph of the Discussion section in lines 400-402 starts with an irritating sentence: "In summary, we conducted this research in order to raise awareness about the fact that reference lists have continued to lengthen in the journal articles of many fields between 1980 and 2019." In the Introduction section, the authors mentioned selected previous studies that already raised the awareness to this fact. Furthermore, the new contribution of this study is testing of the two hypotheses that are mentioned in the end of the Introduction section.

*Response: We appreciate the reviewers’ suggestion. The concluding paragraph has been modified (Line 433-437).*
